# Loss of the Spinocerebellar Ataxia type 3 disease protein ATXN3 alters transcription of multiple signal transduction pathways

**DOI:** 10.1371/journal.pone.0204438

**Published:** 2018-09-19

**Authors:** Li Zeng, Dapeng Zhang, Hayley S. McLoughlin, Annie J. Zalon, L. Aravind, Henry L. Paulson

**Affiliations:** 1 Department of Neurology, Sichuan Provincial People’s Hospital, Chengdu, China; 2 Department of Biology, St. Louis University, St. Louis, Missouri, United States of America; 3 Department of Neurology, University of Michigan, Ann Arbor, Michigan, United States of America; 4 National Center for Biotechnology Information, National Library of Medicine, National Institutes of Health, Bethesda, Maryland, United States of America; University of Minnesota Duluth, UNITED STATES

## Abstract

Spinocerebellar ataxia type 3 (SCA3) is a dominantly inherited neurodegenerative disorder caused by a polyglutamine-encoding CAG repeat expansion in the *ATXN3* gene which encodes the deubiquitinating enzyme, ATXN3. Several mechanisms have been proposed to explain the pathogenic role of mutant, polyQ-expanded ATXN3 in SCA3 including disease protein aggregation, impairment of ubiquitin-proteasomal degradation and transcriptional dysregulation. A better understanding of the normal functions of this protein may shed light on SCA3 disease pathogenesis. To assess the potential normal role of ATXN3 in regulating gene expression, we compared transcriptional profiles in WT versus *Atxn3* null mouse embryonic fibroblasts. Differentially expressed genes in the absence of ATXN3 contribute to multiple signal transduction pathways, suggesting a status switch of signaling pathways including depressed Wnt and BMP4 pathways and elevated growth factor pathways such as Prolactin, TGF-β, and Ephrin pathways. The Eph receptor A3 (*Efna3*), a receptor protein-tyrosine kinase in the Ephrin pathway that is highly expressed in the nervous system, was the most differentially upregulated gene in *Atxn3* null MEFs. This increased expression of *Efna3* was recapitulated in *Atxn3* knockout mouse brainstem, a selectively vulnerable brain region in SCA3. Overexpression of normal or expanded ATXN3 was sufficient to repress *Efna3* expression, supporting a role for ATXN3 in regulating Ephrin signaling. We further show that, in the absence of ATXN3, *Efna3* upregulation is associated with hyperacetylation of histones H3 and H4 at the *Efna3* promoter, which in turn is induced by decreased levels of HDAC3 and NCoR in ATXN3 null cells. Together, these results reveal a normal role for ATXN3 in transcriptional regulation of multiple signaling pathways of potential relevance to disease processes in SCA3.

## Introduction

Spinocerebellar ataxia type 3 (SCA3) is one of nine polyglutamine (polyQ) neurodegenerative diseases caused by an abnormally long polyQ tract in the disease protein, which in SCA3 is ATXN3 [1]. PolyQ diseases are age-related, progressive disorders that typically first manifest in midlife, leading to death 15–30 years later [2–5]. A common neuropathological hallmark in SCA3 and other polyglutamine diseases is the accumulation of ubiquitin-positive nuclear inclusions in neurons [6, 7]. *Atxn3* null mice or *C*. *elegans* do not display obvious neurodegeneration, suggesting that a dominant gain-of-function, rather than a loss-of-function, mechanism principally drives SCA3 pathology, and that normal functions of ATXN3 are not essential [8, 9]. It remains possible, however, that polyQ expansion alters normal functional properties of the disease protein that contribute to the disease process. Many polyQ disease proteins participate in transcription [10], with disease-causing expansions disrupting normal transcriptional profiles in the nervous system. Here we sought to learn more about the potential normal role of ATXN3 in transcriptional regulation.

ATXN3 is a deubiquitinase implicated in multiple aspects of protein quality control associated with the ubiquitin-proteasome system of protein degradation. Its known activities include regulating the action of E3 ligases, participating in proteasomal substrate delivery by interacting with shuttle proteins, and regulating aggresome formation [5, 11–15]. An additional role for ATXN3 in transcriptional regulation is suggested by the ability of ATXN3 to bind DNA and cooperate with transcriptional regulators including CBP, P300, PCAF, TBP, HDAC3, HDAC6 and NCoR [16–20]. In SCA3 disease, polyQ-expanded ATXN3 leads to global transcriptional dysfunction [21–24]. Transcriptome analysis in cerebella from transgenic mice expressing expanded ATXN3 exhibited downregulation of genes involved in glutamatergic neurotransmission, intracellular calcium signaling/mobilization of MAP kinase pathways, GABA_A/B_ receptor subunits, heat shock proteins and transcription factors regulating neuronal survival and differentiation [21]. Conversely, inflammatory related genes and neuronal cell apoptosis related genes are upregulated in expanded ATXN3-expressing cell lines and brain tissue [21–24].

However, whether ATXN3 loss-of-function contributes to SCA3 transcriptional dysfunction is still unknown. To address this hypothesis, we performed oligonucleotide microarray analyses on *Atxn3* wildtype (*Atxn3*-WT) and *Atxn3* knockout (*Atxn3*-KO) mouse embryonic fibroblasts (MEFs). The data show that loss of Atxn3 perturbs the gene expression of several key signaling pathways including Wnt, Prolactin/IL6, TGF-β/BMP4 and Ephrin pathways and cytoskeleton. The most robustly increased transcript was Ephrin-A3 (*Efna3*) which belongs to the family of Ephrins that function in signaling between neurons and have been implicated in Alzheimer’s Disease, amyotrophic lateral sclerosis, and other neurological disorders [25, 26]. Therefore, we chose *Efna3* to investigate further the possible mechanism of gene upregulation occurring in the absence of ATXN3. In *Atxn3*-KO MEFs, increases in the histone deacetylases HDAC3 and NCoR were associated with hyperacetylation of histone H3 and H4 at the *Efna3* promoter, leading to increased *Efna3* transcriptional activity.

## Materials and methods

### Animals

All animal procedures were approved by the University of Michigan Institutional Animal Care and Use Committee and conducted in accordance with the U.S. Public Health Service’s Policy on Humane Care and Use of Laboratory Animals. Genotyping was performed using tail biopsy DNA isolated prior to weaning and confirmed postmortem, as previously described [27]. Atxn3-KO and WT littermate mice, previously described, were sex-and age-matched for each study. Mouse embryonic fibroblasts (MEFs) were isolated as previously described [28]. For biochemical brainstem analysis, animals were euthanized with a lethal dose of ketamine-xylazine and PBS-perfused at 8 weeks of age and tissue was macrodissected for biochemical assessments, as previously described [29].

### Cell culture and treatment

The MEFs from both *Atxn3*-WT and *Atxn3*-KO mice [27] were maintained in DMEM with 10% FBS, 1% Non-essential Amino Acids solution and 1% penicillin/streptomycin at 37° C and 5% CO2. Where needed, cells were treated with Trichostatin A (1μM; Calbiochem), Apicidin (0.1μg/ml; Sigma-Aldrich) or DMSO (Sigma-Aldrich).

### RNA isolation

According to the manufacturer’s protocol, RNA was isolated using RNeasy mini Kits (Qiagen, Valencia, CA) from three biological replicates of *Atxn3*-WT and *Atxn3*-KO MEFs and from five 8-week-old *Atxn3*-KO and *Atxn3*-WT PBS-perfused brainstem extracts, as previously described [29]. Total RNA was quantified on a NanoDrop spectrophotometer, followed by RNA quality assessment on an Agilent 2100 bioanalyzer (Agilent, Palo Alto, CA, USA) for MEF samples and quantitative RT-PCR MEF and brainstem samples.

### Oligonucleotide microarray hybridization and data analysis

For microarray analysis, 10 μg of total RNA in triplicate from *Atxn3*-WT and *Atxn3*-KO samples were submitted to the core facility at University of Michigan. GeneChip mouse genome 430 2.0 arrays (Affymetrix) was used for gene expression analysis of both *Atxn3*-WT and *Atxn3*-KO MEFs. These oligonucleotide arrays contain about 45,101 probe sets of which 37,400 are mouse genes. The detailed array platform information is found at the Affymetrix website (http://www.affymetrix.com/support/technical/libraryfilesmain.affx). Microarray hybridization and image scanning were performed according to the manufacturer’s standard protocols.

Array data was analyzed using the affy, affyPLM, limma packages of the Bioconductor software project implemented in the R statistical environment. Data was normalized with a robust multi-array average (RMA) approach [30]. A weighted linear model was used to detect expression differences between *Atxn3*-WT and *Atxn3*-KO samples [31]. The p-values were adjusted for multiple comparisons using the false discovery rate (FDR) [32]. Only probes with an FDR ≤0.001 and expression difference ≥ 2-fold were considered as statistically significant, which generated 617 gene probes. This set was further cleaned to filter the duplicate probes and any wrongly annotated genes for which we could not identify protein or RNA homologs in the NCBI database; these steps produced a final set of 423 unique genes (GEO ID: GSE117028). To understand the biological significance of this gene set, we made functional annotations based on protein domain information retrieved from the Pfam database [33], and other sequence or motif features, including signal peptides predicted by SignalP [34], transmembrane regions predicted by TMHMM [35], coiled coil regions predicted by COILS [36]. These genes were further classified to a higher hierarchy based on their cellular localizations (i.e. extracellular space, plasma membrane, cytoplasm and nucleus). Key signaling pathways were reconstructed based on functional annotations of proteins according to the domains detected in them and information extracted from the published literature.

### Real-time PCR

Total RNA was prepared using Trizol reagent according to the manufacturer’s protocol (Invitrogen). The cDNA was synthesized in a mixture containing total RNA 1 μg, 1 μl of iScript reverse transcriptase and 4 μl buffer (iScript cDNA synthesis kit, Bio-RAD). Reaction was performed for 5 minutes at 25 °C, 30 minutes at 42 °C and 5 minutes at 85 °C.

Quantitative PCR was performed with SYBR Green Supermix (Bio-RAD) following the manufacturer’s instructions. PCR amplification was performed for 3 min at 95 °C and followed by 40 cycles of 10 s at 95 °C and 30s at 55 °C. PCR amplification of glyceraldehyde-3-phosphate dehydrogenase (GAPDH) mRNA was used as the normalization control. The relative change in mRNA expressions was acquired by the equation: Fold change = 2^-[ΔΔCt]^, ΔΔCt = (Ct _gene of interest_ − Ct _GAPDH_) _KO_ − (Ct _gene of interest_ − Ct _GAPDH_) _WT_. Ct value is the cycle number at which fluorescence signal crosses the threshold. The primers were listed in S1 Table. S2 Table compares the fold changes (*Atxn3*-KO relative to WT MEFs) in microarray and RT-PCR data to illustrate the degree of difference between the assay techniques.

### Constructs and expression plasmids

ATXN3 constructs were maintained in the following vectors: FLAG-ATXN3-Q22 (Addgene; Plasmid ID: 22126), FLAG-ATXN3-Q80 (Addgene; plasmid ID: 22129) in pFLAG. The Efna3 gene promoter reporter constructs were generated in the vector pGL3-Basic (Promega, Madison, WI). Mouse genomic DNA was used as a template to synthesis different lengths of 5’ upstream Efna3. The primers used for amplification were a 3’-reverse primer (TAT GAA GCT TGT TGC TGG TGC ACC) that binds at the transcription start site of Efna3 in conjunction with different 5’-forward primers (Efna3 (-1329): GCACGAGCTCATCAGTCTCTTCCAT CTGGCTT; Efna3 (-569): GCACGAGCTCCCTCTCTGTTTCAGCTGAGATTG; Efna3 (-279): GCACGAGCTCAAGACTCTCCGTCGCTGTC) that bind at different distances of Efna3 promoter. A restriction site for SacI was contained within each forward primer while the reverse primer contained a HindIII restriction site. The PCR products were first subcloned into pCR2.1 vectors (Invitrogen). The constructed pCR2.1 plasmids were then digested with SacI and HindIII and inserted into the multiple cloning region of the pGL3-Basic vector which was digested with the same enzymes. All constructs were verified by sequencing and expression analysis.

### Transient transfection and luciferase reporter assays

MEFs were seeded on 12-well plates. Cell density was 80% confluent on the day of transfection. MEFs were transfected using Lipofectamine LTX and PLUS (Invitrogen) according to the manufacturer’s instructions. 1 μg of indicated luciferase constructs were always co-transfected with 10 ng of the Renilla luciferase plasmid (pRL-CMVvector, Promega). After 24 hours, cells were lysed in 250μl Passive Lysis (Promega), 20μl lysate was added to 100μl LAR II (Promega) for luciferase activity, then 100μl Stop&Glo reagent (Promega) was added to the same well for measuring Renilla luciferase activity. Luciferase activities were measured in the spectraMax M3 microplate reader (Molecular Device, Sunnyvale, CA) using the dual-luciferase reporter assay system (Promega). Data were normalized for activity of Renilla luciferase. Three transfections of each construct plasmids were used in every assay.

### Chromatin immunoprecipitation assay (ChIP)

ChIP assays were performed using the Acetyl-Histone H3 Immunoprecipitation Assay kit (Millipore). 2 X 10^6^ cells of each MEF cell lines (*Atxn3*-WT, *Atxn3*-KO) were fixed with 1% formaldehyde for 10 minutes at 37°C. Cross-linked cells were scraped and lysed with SDS lysis buffer. Cell lysate was sonicated with Bioruptor (Diagenode) to shear DNA to lengths between 200 bp and 600 bp. Indicated antibodies or rabbit IgG were used to immunoprecipitate DNA/protein complexes overnight. After reversal of crosslinks, DNA was purified with PCR purification kit (Qiagen). Real time PCR was conducted on equal concentrations of input and IP derived DNA in triplicate. The primers used for amplification of chromatin fragments of the *Efna3* gene are listed in S3 Table. The amounts of immunoprecipitated chromatin DNA were normalized to input DNA (1% of chromatin DNA). Values are expressed as fold changes over background signals obtained in corresponding IgG controls.

### Antibodies

The following antibodies were used: mouse monoclonal anti-ATXN3 (1:1000; 1H9; Millipore); mouse monoclonal anti-GAPDH (1: 100,000; Millipore); rabbit monoclonal anti-Tubulin-α (1:10,000; Cell Signaling); normal Rabbit IgG (1:1000; Santa Cruz Biotechnology); rabbit polyclonal anti-H3 (1: 5,000; Cell Signaling); rabbit polyclonal anti-H4 (1: 5,00; Cell Signaling); rabbit polyclonal anti-HDAC3 (1:5,000; Abcam); rabbit polyclonal anti-acetyl-Histone H4 (1:5,000; Millipore); rabbit polyclonal anti-acetyl-histone H3 (1:5,000; Millipore); rabbit polyclonal anti-NCoR (1:1000; Millipore); rabbit polyclonal anti-Efna3 (1:1000; Santa Cruz Biotechnology); rabbit polyclonal anti-PCAF (1:500; Millipore); rabbit polyclonal anti-HDAC6 (1:2,000; Millipore); mouse monoclonal anti-p300 (1:2000; Millipore); and peroxidase-conjugated, goat anti-rabbit and goat anti-mouse antibodies (1: 10,000; Jackson Immunoresearch).

### Western blot analysis

Western blot was performed as described previously [27]. Briefly, cell pellets and brain samples were lysed on ice in RIPA buffer (50mM Tris, 150mM NaCl, 0.1% SDS, 0.5% deoxycholic acid, 1% Nonidet P-40, pH 7.4) with Complete Mini Protease Inhibitor tablets (Roche). After sonication and centrifugation, supernatants were supplemented with 1% final SDS and 100mM DTT, boiled and loaded on SDS-PAGE and transferred to polyvinylidene difluoride membrane. Membranes were blocked for 30 min in blocking buffer (TBS-Tween with 5% skim milk), then incubated overnight with primary antibodies at 4°C. Second antibodies were incubated with membranes for 1h at room temperature. Blots were imaged using blue basic autorad film (GeneMate). For semi-quantification, Image-J software was used.

### Statistical analyses

All statistical analyses were carried out using Prism (Graphpad, La Jolla, CA). One-way ANOVA with post hoc comparisons or two-paired *t*-test was applied to compare groups. Results are presented as mean ± SEM. Significant differences were accepted when *p*<0.05.

## Results

### Loss of ATXN3 alters expression of genes principally involved in signal transduction

To understand the contribution of ATXN3 to global gene expression, we utilize genome-wide microarray technique to profile expression differences between *Atxn3*-WT and *Atxn3*-KO MEFs. Analysis of microarray data identified 423 unique genes that were differentially expressed (FDR ≤0.001 and fold change ≥ 2; GEO ID: GSE117028). Of these genes, 270 genes were upregulated, while 153 were downregulated in *Atxn3*-KO MEFs relative to Atxn3-WT MEFs.

**Fig 1:**
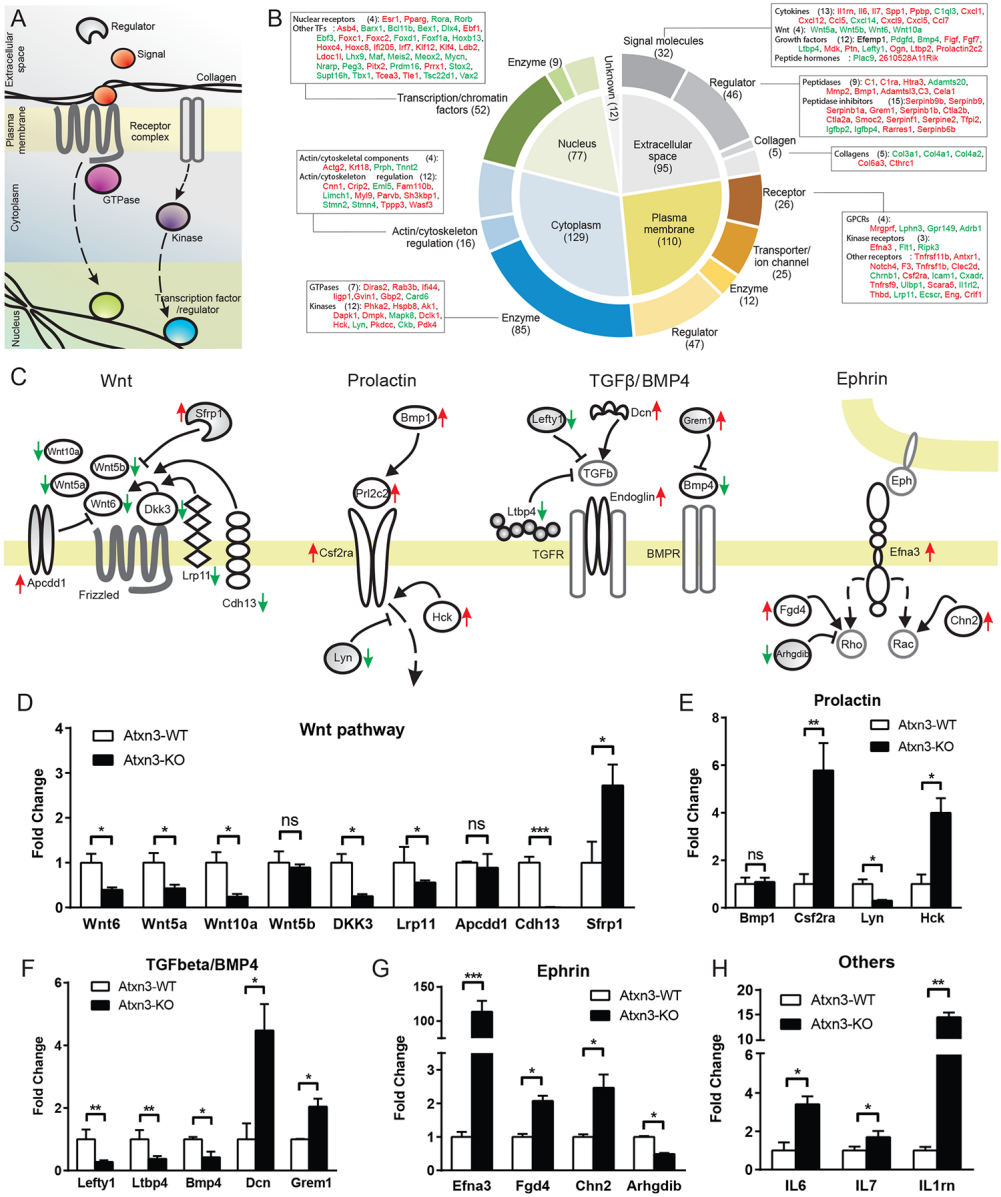
Gene expression changes in *Atxn3*-KO relative to *Atxn3*-WT MEFs. (A) Schematic depicts the major components of cellular signaling pathways that display differential expression in Atxn-3 KO MEFs, including 1) extracellular signal molecules, and their regulators such as peptidases and inhibitors, 2) membrane embedded/bound receptors, transporters and regulators, 3) cytoplasmic signaling enzymes such as GTPases and kinases, 4) and downstream nuclear transcriptional factors and chromatin regulators. (B) Diagram represents the classification of a subset of the 423 differentially expressed genes in *Atxn3*-KO MEFs based on cellular localization and biological function. Dominant functional themes and the major associated genes are listed; up-regulated genes are highlighted in red and down-regulated in green. Numbers in parentheses indicate the total number of genes in each category. (C) The cartoon represents the up- or down-regulation of components of major signaling pathways (Wnt, Prolactin, TGF- β, BMP4 and Ephrin) in MEFs lacking ATXN3. Arrows represent induction or activation whereas blunt-ended lines represent inhibition or repression. Gene expression changes are shown with red or green arrows corresponding to increased or decreased expression. (D-H) Real-time PCR verification of differentially regulated components of four signaling pathways. Among 25 selected genes from the microarray analysis, 22 genes were confirmed to have significant changes in *Atxn3*-KO MEFs relative to WT MEFs. (D) Wnt pathway; (E) Prolactin pathway; (F) TGF-β/BMP4 pathway; (G) Ephrin pathway; (H) Others. Error bars represent the mean ± SD, * *p*<0.05. **p<0.01.

To assess the biological significance of the gene set, we systematically analyzed and functionally annotated the identified differentially expressed genes (Fig 1A–1C). The majority of genes with altered expression in *Atxn3*-KO MEFs are components of various cellular signaling networks. These include: secreted extracellular signaling molecules that initiate transduction cascades (including 13 cytokines, 4 Wnt ligands, 12 other growth factors, and peptide hormones); their regulators (including peptidases critical for ligand maturation); and molecules downstream of the extracellular signaling molecules, namely membrane receptors (including GPCRs, receptors with kinase activities), cytoplasmic signal transducers (including GTPases and kinases), and nuclear transcriptional factors and chromatin regulators (including 4 nuclear receptors and 48 other TFs with diverse DNA-binding domains) which are likely to function as effectors of gene expression (Fig 1). The main effect of the loss of ATXN3 appears to be significant changes in multiple cellular signaling pathways.

We inferred molecular interactions between the identified gene products to reconstruct key affected signaling pathways (Fig 1C) including the Wnt, prolactin, TGF- β/BMP4, and ephrin signaling pathways. We predicted that differential expression of these genes should either positively or negatively impact signaling transduction, leading to different outputs from these signaling pathways. For example, in cells lacking ATXN3 we could infer that the Wnt pathway is depressed due to downregulation of four Wnt ligands and three important positive regulators, namely Lrp11, Dkk3, and Cdh13, and upregulation of two inhibitors, namely Sfrp1 and Apcdd1 (Fig 1D). In contrast, both Prolactin (Fig 1E) and Ephrin (Fig 1G) signaling are expected to be elevated due to increased expression of their ligands, receptors and positive regulators, and decreased expression of their inhibitors. We found differential regulation of the signaling pathways of different members of the TGFβ family (Fig 1F) (e.g. Lefty1 and BMP4): Lefty, the inhibitor of TGF-β, is down-regulated along with another unrelated inhibitor of this pathway LTBP4. However, decorin (DCN), a proteoglycan with leucine-rich repeats that binds and positively regulates TGFβ proteoglycan, and the TGFβ co-receptor endoglin are up-regulated. In contrast, BMP4 is down-regulated whereas its inhibitor gremlin1 (GREM1) is up-regulated. This would suggest that TGFβ signaling is elevated in *Atxn3*-null MEFs whereas signaling by BMP4 might be depressed. Many other differentially expressed genes are implicated in additional signaling networks such as those initiated by cytokines (e.g. IL6, IL7, Il1rn)(Fig 1H). Alterations in these signaling pathways represent a snapshot of the changes to global signaling activity precipitated by loss of ATXN3.

We also noted changes in the expression of genes encoding structural proteins or regulators of the extracellular matrix (ECM) and cytoskeleton. Changes related to the ECM include dysregulation of five collagens that are primary components of ECM, extracellular peptidases that are responsive to the breakdown and remodeling of extracellular matrix (e.g. MMP2, complement component 1, and complement component 3), and many secreted peptidase inhibitors that regulate peptidase activities. Changes in the cytoskeletal organization include dysregulation of the cytoskeletal components actin γ2, keratin 18, peripherin, troponin T2, and 12 other genes whose products are involved in regulation of cytoskeleton structure and dynamics (Fig 1B). This result suggests that loss of ATXN3 is associated with the remodeling or disorganization of both ECM and intracellular cytoskeletal structures. It is not clear if these changes are downstream of the above-noted changes in signaling pathways.

### Loss of Efna3 promoter repression in Atxn-3 KO cells

**Fig 2:**
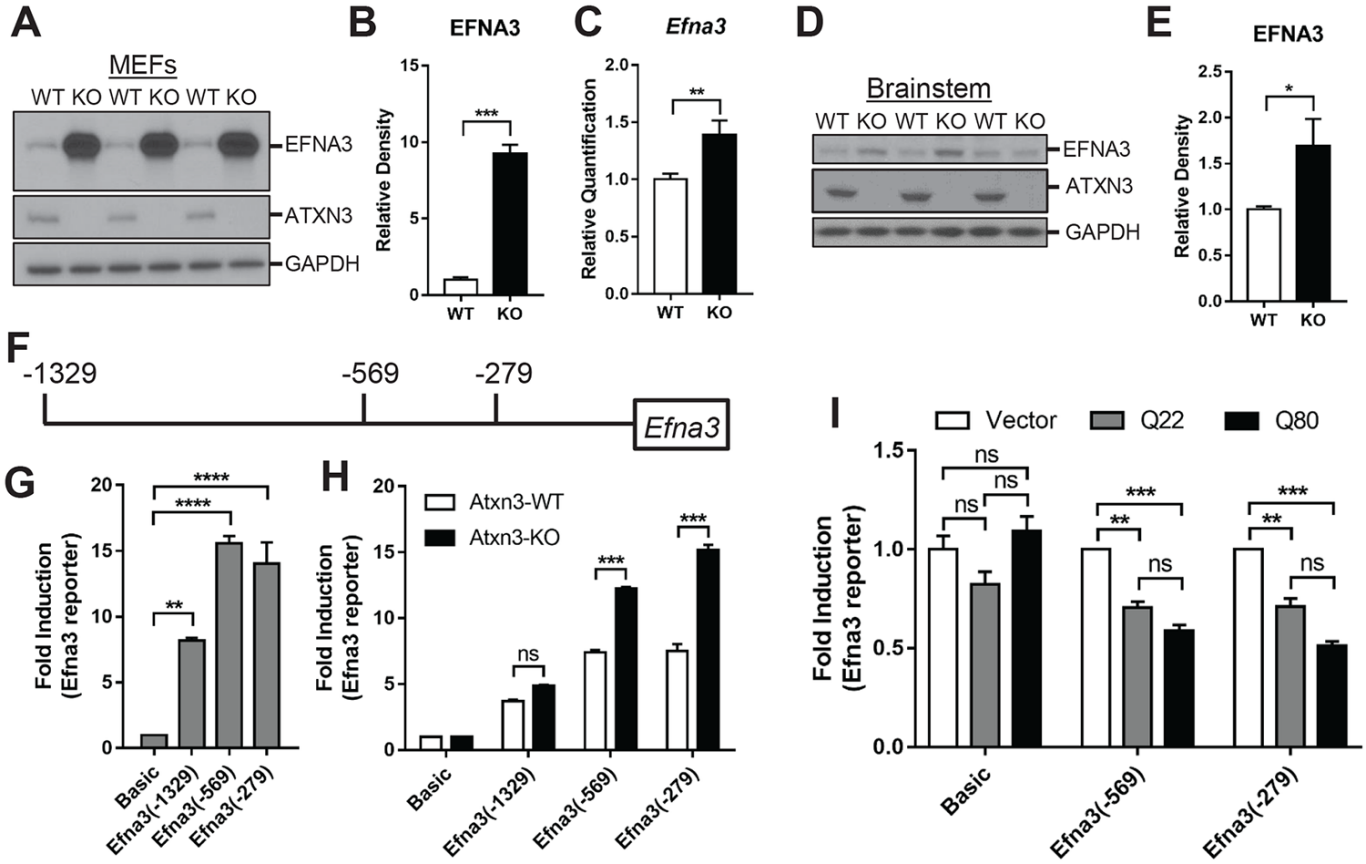
Loss of ATXN3 induces *Efna3* upregulation. (A, B) Western blotting and quantitative analysis show increased expression of EFNA3 protein in *Atxn3*-KO MEF cells (n = 3 per genotype). (C) Quantitative RT-PCR of *Efna3* shows elevated transcript in *Atxn3*-KO mouse brainstem samples. (D, E) Representative western blotting and quantitative analysis show increased expression of EFNA3 protein in 8-week-old *Atxn3*-KO brainstem lysates (n = 5 per genotype). (F) Diagram shows *Efna3*-luciferase reporter gene constructs containing different lengths of the *Efna3* promoter. (G) Dual luciferase reporter assays of WT and *Atxn3*-KO MEFs transfected with constructs containing *Efna3* promoter of different lengths. The luciferase activities are presented as fold change normalized to levels in cells transfected with pGL3-Basic vector. (H) All three promoter constructs mediate transcription in WT and *Atxn3*-KO MEF cells, with transcription of *Efna3* significantly increased in the absence of ATXN3. (I) Reporter assays of transiently transfected *Atxn3*-KO MEFs using pGL3-Basic vector, pGL3-Efna3 (-569) or pGL3-Efna3 (-279) co-transfected with an expression plasmid encoding normal ATXN3 (Q22) or expanded ATXN3 (Q80). Reporter activities are presented as fold change from that measured in cells transfected with empty vector (results averaged from three independent experiments). Overexpression of normal ATXN3 (Q22) or expanded ATXN3 (Q80) suppresses transcription from the *Efna3* promoter. Error bars represent the mean ± SEM. *p<0.05, ** *p*<0.01, *** *p*<0.001, ^ns^ *p*>0.05.

To explore the molecular mechanism by which ATXN3 participates in transcriptional regulation, we focused on Eph receptor A3 (Efna3) since Efna3 is a key component of ephrin signaling and displayed the highest transcriptional increase in *Atxn3*-KO MEFs (Fig 1). Western blot analysis was performed to detect the expected change in EFNA3 protein level resulting from altered mRNA expression in *Atxn3*-KO cells. Relative to WT MEFs, EFNA3 was significantly increased in *Atxn3*-KO MEFs (Fig 2A and 2B). We further documented changes in Efna3 in *Atxn3*-KO mouse brainstem tissue: again, there was an increase both in transcript (Fig 2C) and protein levels (Fig 2D and 2E) relative to levels in wildtype mice at 8 weeks of age.

By comparing upstream genomic regions of human and mouse *Efna3* genes, we identified conserved regions that could serve as the *Efna3* promoter to mediate transcriptional repression or activation. Three constructs containing different lengths of the 5’ region of *Efna3* promoter (from position of -1329bp, -569bp, or -279bp to the transcription initiation site) were fused to the luciferase pGL3 reporter gene (Fig 2F), and transiently transfected in HEK293 cells. All three constructs induced transcription (Fig 2G), but constructs harboring the *Efna3* gene promoter region extended to -569 and -279bp displayed the strongest effect. This result indicates that the conserved upstream region is indeed the promoter of *Efna3* gene, consistent with a previous report [37].

We next examined if the apparent transcriptional repression by ATXN3 on the *Efna3* gene is mediated by this promoter region. When transiently transfected into WT and *Atxn3*-KO MEFs, all three promoter constructs resulted in activation, with the level of activation being consistently higher in *Atxn3*-KO cells expressing -569bp and -279bp constructs relative to *Atxn3*-WT cells (Fig 2H). These results indicate that ATXN3 represses *Efna3* transcription by acting, either directly or indirectly, on the promoter region.

We further tested whether transiently expressed normal human ATXN3 (Q22) or pathological ATXN3 (Q80) could repress transcription of the Efna3(-569) or Efna3(-279) reporter constructs in *Atxn3*-KO cells. Both normal and expanded ATXN3 significantly repressed Efna3 transcription by approximately 20% but had no effect on expression of the control pGL3 luciferase vector (Fig 2I). These findings support a normal repressive action of ATXN3 at the *Efna3* promoter that persists in the presence of polyglutamine expansion.

### Loss of ATXN3 induces acetylation of histones at *Efna3* promoter region

**Fig 3:**
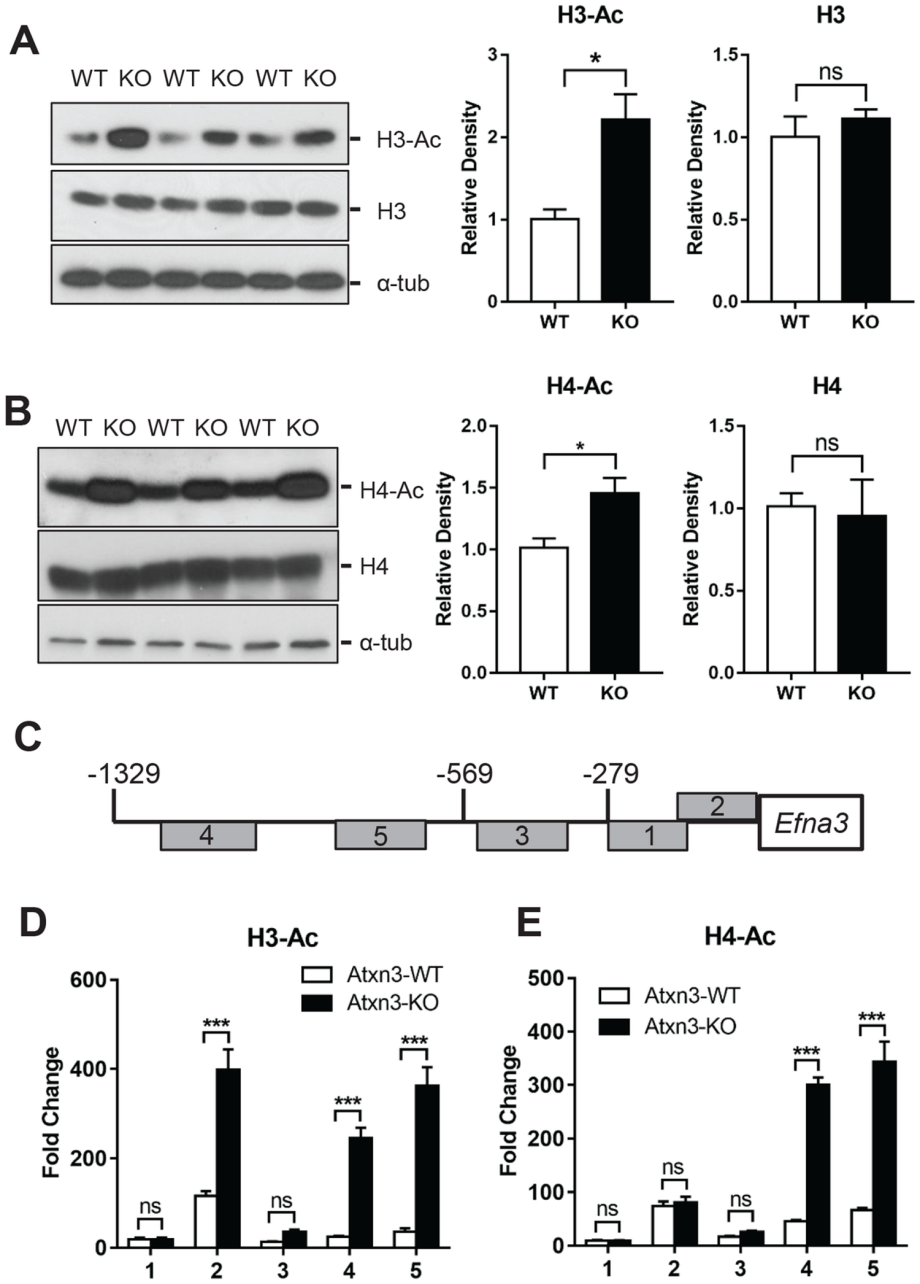
H3 and H4 are hyperacetylated in *Atxn3*-KO MEFs. (A-B) Western blot and quantitative analysis demonstrate that absence of ATXN3 increases levels of acetylated H3 and H4. Blots were quantified with Image J (two-paired t-test, n = 3). Results are means ± SEM. * *p*<0.05, ** *p*<0.01, *** *p*<0.001, ^ns^ *p*>0.05. (C) The schematic of PCR amplicons locations in the *Efna3* gene promoter (1–5) analyzed by ChIP assay. (D-E) H3 and H4 are hyperacetylated in *Efna3* promoter regions in *Atxn3*-KO cells. ChIP assays of 5 adjacent promoter regions (1–5), using chromatin from WT and *Atxn3*-KO cells and antibodies against acetylated H3 (D) acetylated H4 (E). Fold change for each antibody was calculated as the ratio of immunoprecipitated chromatin DNA over total input chromatin DNA, normalized to control IgG. Results were averaged from three independent experiments. Error bars represent mean ± SEM.

Previous studies have suggested potential mechanisms by which ATXN3 regulates transcription, including direct binding to DNA sequences, interacting with transcriptional regulators, and modulating histone acetylation [16, 24, 38, 39]. Because loss of ATXN3 induced widespread gene expression changes and histone acetylation/deacetylation is known to be a global regulator of gene expression, we tested the potential role of histone acetylation in mediating the upregulation of *Efna3* expression in *Atxn3*-KO MEFs. We first tested whether global acetylation of Histone H3 and Histone H4 is increased in *Atxn3*-KO MEF cells. By western blot, we observed an approximately 100% and 50% increase in acetylated-H3 and H4, respectively, in *Atxn3*-KO cells relative to WT cells with no change in total H3 and H4 expression levels (Fig 3A and 3B). To determine if this increased histone acetylation directly impacts *Efna3* promotor expression, we performed chromatin immunoprecipitation (ChIP) assays on chromatin from WT or *Atxn3*-KO MEFs, using specific antibodies against acetylated histone H3 and H4 and amplifying five adjacent *Efna3* promoter regions (Fig 3C). In *Atxn3*-KO MEFs, increased acetylated histone H3 was observed at promoter regions 2, 4 and 5 (Fig 3D), and increased acetylated histone H4 was observed at promoter regions 4 and 5 (Fig 3E). Based on these results obtained for one of the differentially expressed genes from our original microarray analysis, we suggest that the gene expression changes induced by loss of ATXN3 are at least partly mediated by increased histone acetylation.

### ATXN3 alters histone acetylation via a pathway containing HDAC3 and NCoR

**Fig 4:**
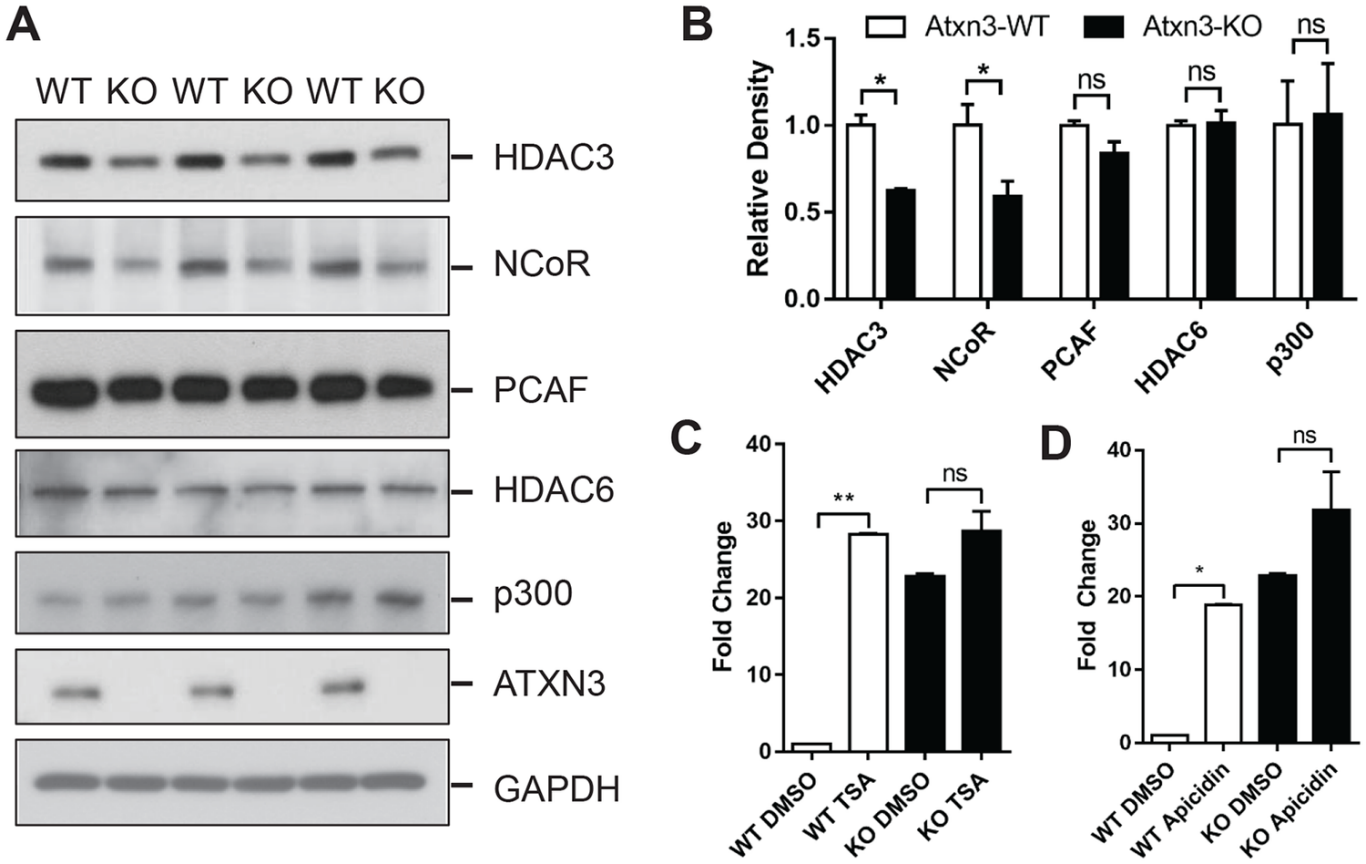
Loss of ATXN3 is associated with decreased levels of HDAC3 and NCoR in MEFs. (A) Western blot analysis of HDAC3, NCoR, PCAF, HDAC6, p300, ATXN3 and GAPDH in lysates from WT and *Atxn3*-KO MEFs. (B) Semiquantitative analysis shows the decrease in HDAC3 and NCoR in *Atxn3*-KO cells. Blots were quantified with Image J (two paired t-test, n = 3). Results are means ± SEM, * p<0.05. (C-D) HDAC inhibitors TSA and Apicidin induce transcriptional upregulation of *Efna3* gene. WT and *Atxn3*-KO cells were treated with TSA (1μM) (C) or Apicidin (0.1μg/ml) (D) for 24 hours, RNA was isolated and RT-PCR analysis was performed to assess *Efna3* expression. Results are expressed as fold change from control. Error bars represent mean ± SEM (n = 3). * *p*<0.05, ** *p*<0.01, ^ns^ *p*>0.05.

We next sought to identify the likely mechanism of increased histone acetylation induced by the loss of ATXN3. The histone acetylation state of a given chromatin locus is controlled by two classes of antagonizing histone-modifying enzymes, histone acetyltransferases (HATs) and deacetylases (HDACs), which add or remove acetyl groups to/from target histones [40]. Interestingly, several HATs (including CBP, P300, PCAF), HDACs (including HDAC3, HDAC6), and the transcriptional corepressor NCoR which recruits HDACs to the promoter region, are reported to interact with ATXN3 [16, 17, 41–43]. To identify which histone acetylases and regulators are likely involved in establishing the histone acetylation and enhanced gene expression of *Efna3* induced by the loss of ATXN3, we measured their nuclear protein levels in both *Atxn3*-KO and WT cells. Levels of HDAC3 and NCoR are significantly decreased in *Atxn3*-KO cells, whereas no difference was found with other regulators including p300, PCAF, HDAC6 (Fig 4A and 4B). Furthermore, 24-hour treatment of MEF cells with the broad HDAC inhibitor Trichostatin A (TSA) at 1μM (Fig 4C) or the HDAC3 specific inhibitor Apicidin at 0.1μg/ml (Fig 4D) resulted in greatly upregulated mRNA level of *Efna3* gene by 20~30 fold in WT MEFs, but not in in *Atxn3*-KO MEFs. These findings support the model that ATXN3 modulates HDAC complexes, such as HDAC3 and NCoR levels, to alter the acetylation state of histones and ultimately dictate gene expression status in *Atxn3*-KO MEF cells.

## Discussion

Here we have shown that loss of ATXN3 in MEFs results in widespread gene expression changes affecting many signal transduction pathways. We validated many of these transcriptional changes and defined a potential mechanism for differential expression of *Efna3*, a major component of the ephrin signaling pathway. *Efna3* was the most elevated transcript in *Atxn3*-KO MEFs relative to wildtype controls and was also found to be elevated in *Atxn3*-KO mouse brainstem, a selectively vulnerable region in SCA3. Our results in MEFs further show that this marked *Efna3* upregulation in the absence of ATXN3 correlates with enhanced acetylation of histone H3 and H4. Additional evidence supports the view that at least two histone acetylation regulators, HDAC3 and NCoR, are responsible for modulating the acetylation state of these histones in the presence of ATXN3. Together, our findings suggest that ATXN3 normally functions to regulate histone acetylation through histone deacetylases, including HDAC3 and NCoR, and thereby regulate the transcription of a large set of genes. The results warrant further assessment of the role that loss of ATXN3 transcriptional regulation may play in SCA3 disease pathogenesis.

### ATXN3 and signal transduction pathways

A previous study by Rodrigues and colleagues [8] found significant deregulation of signal transduction in *Atxn3*-KO C. *elegans*, including 2 serine/threonine specific protein phosphatases, 8 protein-tyrosine phosphatases, 6 serine/threonine kinases, 3 casein kinase and others. However, the authors did not construct affected signaling pathways based on these enzymes [8]. Here we took a basic approach to assess the role of ATXN3 in regulating gene expression: transcriptional profiling in *Atxn3*-KO MEFs relative to WT control MEFs. While MEFs arguably are of limited relevance to SCA3 brain pathology and may highlight pathways that are specific or particularly relevant to this cell type, their homogenous composition relative to brain tissue provided us an opportunity to gauge the transcriptional effects of eliminating ATXN3. Similar to the C. *elegans* study, we identified several signaling pathways (e.g. Wnt, TGFβ/BMP4, and Ephrin) that were significantly altered in *Atxn3*-KO MEFs and are implicated in diseases of the nervous system including neurodegenerative disorders. Specifically, Wnt signaling regulates several developmental processes, including neurogenesis and synaptic differentiation [44] and has been shown to be neuroprotective in HD and Alzheimer’s disease (AD) [45–47]. Similarly, TGFβ levels are altered in cortical neurons and peripheral blood of HD patients and may also place a role in AD and Parkinson’s disease [48, 49]. Additionally, recent studies of disrupted Eph/ephrin signaling which regulates synaptic function has been linked to AD pathogenesis [25, 26]. Taken together, bioinformatics analysis of the genes commonly altered in our study show enrichment for categories associated with pathways affected in neurodegenerative diseases. Interestingly, several of the genes we identified, namely IL-1ra, IL-6, C1s, MMP-2, PPAR gamma, Csf2ra and many extracellular cytokines, peptidases, peptidase inhibitors, were previously found to be differentially expressed in SCA3 disease-relevant models, including rat cell lines expressing expanded ATNX3 versus non-expanded ATXN3 [23] and human blood samples from SCA3 patients versus unaffected individuals [50]. Thus, the status change of signal transduction pathways associated with loss of ATXN3 is potentially relevant to SCA3 pathogenesis. Further molecular studies in additional disease-relevant model systems will be necessary to elucidate the extent to which the deleterious actions of mutant, expanded ATXN3 reflect a loss of normal function in transcriptional regulation.

### ATXN3 and transcriptional regulation

There are several potential mechanisms by which ATXN3 may regulate transcription. First, ATXN3 is transported into the nucleus, particularly in the disease state [51], where it binds histone proteins and cooperates with numerous transcriptional regulators including CBP, P300, PCAF, TBP, HDAC3, HDAC6 and NCoR [16–20] to control transcription of target genes. Second, through its deubiquitinase (DUB) activity ATXN3 can regulate transcription factors and repressors by directly altering ubiquitin chains attached to such proteins, thereby affecting their behavior or degradation [8]. Third, polyglutamine expansion may directly affect functional properties of ATXN3 and its normal protein interactions, which could alter gene expression in numerous ways. A direct interaction of ATXN3 with HDAC3 and NCoR has previously been established in SCA3 cell lines and human brain extracts yet with only normal (i.e. not expanded) ATXN3 being associated with increased deacetylase activity and repression of gene expression [17]. Our study also supports a role for ATXN3 in regulating histone deacetylase expression: in the absence of ATXN3 in MEFs, we found significant decreased expression of two HDACs, HDAC3 and NCoR, that led to hyperacetylated histone states.

A limitation to our study is that we did not profile how mutant ATXN3 affects histone deacetylase expression levels and therefore cannot be certain that the transcriptional dysfunction seen in *Atxn3*-KO cells also occurs in mutant ATXN3-expressing cells. Due to the robust increase in *Efna3* expression in *Atxn3*-KO cells as a direct result of hyperacetylation to the *Efna3* promotor region, one interesting future direction will be to assess the acetylation state of *Efna3* expression in disease models, specifically in the context of different *Efna3* promotor regions. Our data in Fig 2 shows significantly greater *Efna3* reporter induction in *Atxn3*-KO cells for the -569 and -279 constructs, but not for the -1329 construct, that may reflect global histone changes repressed by the upstream Efna3 promoter between -1329 to -569 region in this overexpression system. Further investigation into the regulatory elements of the *Efna3* promoter will be required for a full understanding of *Efna3’s* potential role in SCA3 disease. Importantly, our study also shows increased expression of Ephrin-A3 at both the RNA and protein level in the brainstem of *Atxn3*-KO mice, supporting the view that the absence of ATXN3 leads to dysregulation of this signaling pathway in a disease-relevant brain region and suggesting that Ephrin-A3 is a compelling candidate for further study in SCA3 disease model systems. Other altered signaling pathways highlighted by our study represent additional candidates for study in disease-relevant brain regions to clarify the potential role of partial loss of disease protein function in SCA3. In addition, a thorough temporal assessment of gene changes in *Atxn3*-null tissues may shed light on the normal functions of ATXN3 during development and aging and the potential implications of ATXN3 loss of function over time.

Finally, this study does not investigate the contribution of AXTN3’s DUB activity to regulating gene expression. For example, ATXN3 has been shown to modulate transcription factor and repressor degradation by directly altering ubiquitin chains attached to such proteins [5, 20]. ATXN3 also directly regulates the ubiquitination state of HDAC3 to modulate IFN-I antiviral activity during viral infection [52]. Future studies employing catalytically inactive ATXN3 will be needed to determine whether the transcriptional changes observed in our study reflect loss of DUB activity rather than loss of other, less well-established functions of the protein.

## Supporting information

S1 Table List of primer sequences used for real-time PCR in Fig 1. (PDF)

S2 Table Comparison of microarray and real-time PCR (RT-PCR) fold change of differentially regulated components of signaling pathways. 22 of the 25 differentially expressed microarray genes were confirmed to have significant changes in *Atxn3*-KO MEFs relative to WT MEFs by RT-PCR (red shading = upregulated; green shading = downregulated). n.s. = not significant; * *p*<0.05, ** *p*<0.01, *** *p*<0.001, **** *p*<0.0001.
(PDF)

S3 Table List of primers sequences used for Efna3 gene promoter PCR amplicons locations (1–5) as analyzed by ChIP assay. (PDF)
