# Supplementary material for: Loss of the Spinocerebellar Ataxia type 3 disease protein ATXN3 alters transcription of multiple signal transduction pathways
Source: PLoS One. 2018 Sep 19;13(9):e0204438. doi: 10.1371/journal.pone.0204438 (PMC6145529; doi:10.1371/journal.pone.0204438)
Supplement: S1 Table — (PDF) [file pone.0204438.s001.pdf]

Gene	Forward	Reverse
sfrp1	CAGCTTTTGAACTGGCCACC	CAAAGTGAGGCTGTTGGCAC
Apcdd1	AGCCTCCCTCCTCAATGTCT	CTCGGGTGTCTTGCTCCATT
wnt10a	CGAGAGCCTCACAGAGACATC	GTGGCATTTGCACTTACGCC
wnt5a	AAAGGGAACGAATCCACGCT	CAGCACGTCTTGAGGCTACA
wnt5b	GGGAGAGACAGTGTGGAAGTC	AACATCTTCCAAAGCGGAGC
wnt6	TCTGGGCCATCTACAGGACT	GGCTGGTGTAAACCCCAAGT
dkk3	GACACTCAGCACAAACTGCG	CTCCGTGCTGGTCTCATTGT
lrp11	GCGCCAGAGAGTCAGATCAA	GAGTCGGCATGTCACCATGA
Cdh13	GACCTTCACCCCAACACAG	GGTTGTAGTTTGCCTTGTTCAG
bmp1	GAACTGGAGTCCTGCTTCCC	CCCCAAGGAGGAATGGTCAC
csf2ra	CAGTGCATTGCGAACGACTT	GAAGAGTTACAGGACGCGGT
hck	TTGCTGACTTTGGACTGGCA	CCATGCTCTAGTGCCCGAAT
lyn	GCTCCAGAGGCCATCAACTT	ATCTGCGTTGGTTCTCCCTG
lefty1	CTCAGATGGGGCGCTCATAC	ACACACAGGGATTGCCCATT
Dcn	CACAGAAGCGGTAACGAGCA	GTCCAGCCCAAGAGACTTGT
Ltbp4(a,b)	TTCAGTGCGTCTGTGACCAA	GGAACACAGCGTCCAGTCAT
Grem1	GACAGAATGAATCGCACCGC	TGGCTCCTTGGGAACCTTTC
bmp4	CGGAAGCTAGGTGAGTTCGG	AGAATCCCATCAGGGACGGA
Efna3	ACTTGAAGATGACCCAGAAGC	CCCAGAGAACAATCCCGTAAC
Fgd4	CAGCGAGAGCGACCATAACT	CACAAGTATTGATGCCTGCGG
Arhgdib	GGAGCCTGGAATAGAGCAAT	GACTCTCGTCATCCTTGTCCA
chn1	AGCATCTGGAGGGCTTTCCT	ACCGGTCAATGGTGCTATGA
chn2	ATGTGCTCTCAGGAACTGTGG	CCTTTGAGCCCCACCTTGAC
IL7	ATTATGGGTGGTGAGAGCCG	ATGTGACAGGCAGCAGAACA
IL1rn	CCTGGGTGCTCCTTTATACAC	CATCTTGCAGGGTCTTTTCC
IL6	CGGCCTTCCCTACTTCACAA	TCTGCAAGTGCATCATCGTT

S1 Table. List of primer sequences used for Real-Time PCR in Fig 1.
