# Supplementary material for: Loss of the Spinocerebellar Ataxia type 3 disease protein ATXN3 alters transcription of multiple signal transduction pathways
Source: PLoS One. 2018 Sep 19;13(9):e0204438. doi: 10.1371/journal.pone.0204438 (PMC6145529; doi:10.1371/journal.pone.0204438)
Supplement: S2 Table — 22 of the 25 differentially expressed microarray genes were confirmed to have significant changes in Atxn3-KO MEFs relative to WT MEFs by RT-PCR (red shading = upregulated; green shading = downregulated). n.s. = not significant; * p<0.05, ** p<0.01, *** p<0.001, **** p<0.0001. (PDF) [file pone.0204438.s002.pdf]

Gene	Microarray KO/WT (Fold change)	RT-PCR KO/WT (Fold change)	Significance (RT-PCR)
Sfrp1	2.68	2.75	*
Apcdd1	1.32	-1.12	n.s.
Wnt10a	-1.01	-4.15	*
Wnt5a	-1.08	-2.33	*
Wnt5b	-1.43	-1.12	n.s.
Wnt6	-1.54	#DIV/0!	**
Dkk3	-2.19	-4.00	*
Lrp11	-1.48	-1.79	*
Cdh13	-2.51	-170.34	***
Bmp1	1.41	1.08	n.s.
Csf2ra	1.65	5.78	**
Hck	1.92	3.99	*
Lyn	-1.4	-3.31	*
Lefty1	-1.75	-3.60	**
Dcn	1.92	4.48	*
Ltbp4(a,b)	-1.04	-2.67	**
Grem1	1.54	2.05	*
Bmp4	-1.22	-2.36	*
Efna3	6.11	#DIV/0!	***
Fgd4	1.16	2.07	****
Arhgdib	-1.26	-2.05	**
Chn2	1.03	2.47	*
IL7	1.02	1.69	*
IL1rn	3.06	14.44	**
IL6	1.03	3.40	**

S2 Table. Comparison of microarray and real-time PCR (RT-PCR) fold change of differentially regulated components of signaling pathways. 22 of the 25 differentially expressed microarray genes were confirmed to have significant changes in *Atxn3*-KO MEFs relative to WT MEFs by RT-PCR (red shading=upregulated; green shading=downregulated). n.s.=not significant; * $p<0.05$, ** $p<0.01$, *** $p<0.001$, **** $p<0.0001$.
