# Supplementary material for: Loss of the Spinocerebellar Ataxia type 3 disease protein ATXN3 alters transcription of multiple signal transduction pathways
Source: PLoS One. 2018 Sep 19;13(9):e0204438. doi: 10.1371/journal.pone.0204438 (PMC6145529; doi:10.1371/journal.pone.0204438)
Supplement: S3 Table — (PDF) [file pone.0204438.s003.pdf]

No	Forward	Reverse
1	AGC ACA TCT TCT GGC TTT CCT AC	GTC AAG TTT GAC ACT GGA GAT AAG G
2	GAG CGG AGC ATG GTA ACT TC	CTG GCT TAC CTT CGT TGG AA
3	CTA AGA GGA CTG TGA ACA CAC AGA A	GAA GGC AGC TAT TTG TTT GCT ATA C
4	CGT TAA CGT TGT CAA CCT AAT TTT C	TTC AGC CAT GTA ATT TAT CCA ATT T
5	GAG TTA ATC TCC GAG TCT CAC AAA A	CCA TAG CTT CAG ACT ACC ACT CAT T

S3 Table. List of primers sequences used for Efna3 gene promoter PCR amplicons locations (1-5) as analyzed by ChIP assay.
